# Malignant Transformation of Oral Epithelial Dysplasia in Southern Finland

**DOI:** 10.1002/hed.70241

**Published:** 2026-03-26

**Authors:** Katariina Leino, Hanna K. Laine, Ville Kinnula, Jaana Rautava

**Affiliations:** ^1^ Department of Oral and Maxillofacial Diseases University of Helsinki Helsinki Finland; ^2^ Biostatistics Center Helsinki University Hospital, University of Helsinki Helsinki Finland; ^3^ Department of Pathology, Helsinki University Hospital HUSLAB Diagnostics Helsinki Finland

**Keywords:** dysplasia, malignant transformation, malignant transformation time, oral cavity, risk factor

## Abstract

**Background:**

Oral epithelial dysplasia (OED) often precedes oral squamous cell carcinoma (OSCC). The aim was to elucidate the risk and time of malignant transformation (MT) of OED.

**Methods:**

Patients diagnosed with OED between 2001 and 2011 in Helsinki and Uusimaa, Finland were included. Kaplan–Meier and Cox proportional hazard analysis factors associated with the MT time.

**Results:**

Dysplasias of 225 patients (mean age 60.0 years, 51.6% females) were diagnosed as mild (85.8%), moderate (11.6%), or severe (2.7%). MT occurred in 14.7% (51.5% females, 48.5% males) at a median time of 5.5 years (IQR 1.7–12.5 years). MT occurred in 33.3% of severe (*n* = 6), 34.6% (*n* = 26) of moderate, and 11.4% (*n* = 193) of mild dysplasia. Dysplasia severity was associated with an increased risk for MT.

**Conclusions:**

MT of OED was 14.7%. The severity of OED is a predictor for MT.

## 1 Introduction

Over 90% of oral cavity cancers are oral squamous cell carcinomas (OSCC) [1, 2, 3]. In recent years, OSCC incidence has increased among younger age groups and females [4]. However, the incidence rate remains higher with males than females [4], and during the past 10 years, the incidence has increased in both sexes; in males by 2.2% and in females by 0.9% [2]. In Finland, there are approximately 400 new OSCCs per year [5].

An estimated 80% of OSCCs are preceded by oral potentially malignant disorder (OPMD) [6], described commonly as leukoplakia, erythroplakia, erythroleukoplakia, lichen planus, or lichenoid reaction [7]. A biopsy is taken from OPMD to detect oral epithelial dysplasia (OED). In OED, the normal maturation of oral epithelium is disturbed, leading to an increased risk of OSCC compared with healthy epithelium. OED carries the same risk factors as OSCC, of which smoking and alcohol consumption are the most significant risk factors, with the risk being dose related [3, 6].

Traditionally, OED has been graded into three categories: mild, moderate, and severe. The grading is based on the proportion of architectural and cytological alterations in epithelium thickness. With an aim to increase the objectivity of OED diagnosis, the World Health Organization (WHO) has suggested grading OED into two grades, low‐ and high‐risk dysplasia [8]. In addition, a new category of differentiated dysplasia has been suggested for grading purely architectural abnormalities without cytological changes [9, 10].

The malignant transformation (MT) rate for OED varies between 6.6% and 36.4% [9]. MT occurs most likely 2–5 years after OED diagnosis [9]. Sex or age has not been reported to influence the risk of MT [9]. In Finland, we have previously reported MT from OED to OSCC to occur in 7.5% of patients in a mean time of 5.2 years (range 0.7–29 years) in southwestern Finland [9]. OSCC most likely develops in the same place as OED [9]. The aim of this study was to further define the risk and time span of MT of OED in southern Finland, the largest hospital district of Finland.

## 2 Methods

The records of patients with a histopathological diagnosis of OED in years 2001–2011 were retrospectively retrieved from the registry of the Department of Pathology, HUSLAB Diagnostics, University of Helsinki, Helsinki, Finland. The study protocol was approved by the Helsinki University Hospital area in southern Finland (HUS/124/2023) and the HUS Regional Medical Research Ethics Committee (6349/2024).

The study comprised patients with an OED diagnosis collected from the pathology registry of HUSLAB between January 1, 2001, and December 31, 2011. If a patient had several OED samples and diagnoses during the study period, the timewise first sample was included in this study. If more than one sample was registered under the same sample number, the one with the most severe dysplasia was included in the study. Patients with any previous cancer in the head and neck region were excluded. In addition, patients who developed carcinoma in the head or neck region within 6 months of their first biopsy were excluded to avoid pre‐existing carcinomas.

During the period of 2001–2011, OEDs were categorized using a three‐grade system. OED diagnoses were collected from the pathologists' reports. An OED diagnosis in this study is the original diagnosis that has been signed out by a HUSLAB pathologist. The OED diagnoses have not been re‐evaluated. An OED diagnosis is subjective in nature, but with this premise, our aim is to represent the real‐life situation. A diagnosis of differentiated dysplasia was not yet presented during our study period 2001–2011 and is therefore not represented in this study.

Patient information was collected from the biopsy sample referral letters and supplemented from the Apotti patient database. Apotti is a large‐scale electronic health system that integrates the records of social and health care services in the Helsinki Metropolitan area. Sex, age at the time of OED diagnosis, location of the biopsy, size and appearance of the clinical finding, tobacco, snuff, and alcohol consumption, other diseases and medications or known genetic conditions and possible death, and/or OSCC diagnosis dates were collected for the study. A Finnish social security number was used to fill in missing information regarding the abovementioned information collected from the Apotti patient database. Follow‐up time was considered from the date of index biopsy to either the date of OSCC diagnosis or death or, if neither occurred, the follow‐up ended on November 30, 2024.

Statistical analysis was performed using IBM SPSS Statistics software for Windows version 29.0, and R version 4.5.1 was used for visualization. The cumulative probability of MT was described using Kaplan–Meier survival estimates and their 95% confidence intervals at 5 years of follow‐up. Log‐rank tests and univariate Cox proportional hazard analysis were undertaken to explore the factors associated with the time of transformation to OSCC. The proportional hazards assumption was verified by a visual inspection of the log–log survival plot.

## 3 Results

### 3.1 Patients

**FIGURE 1:**
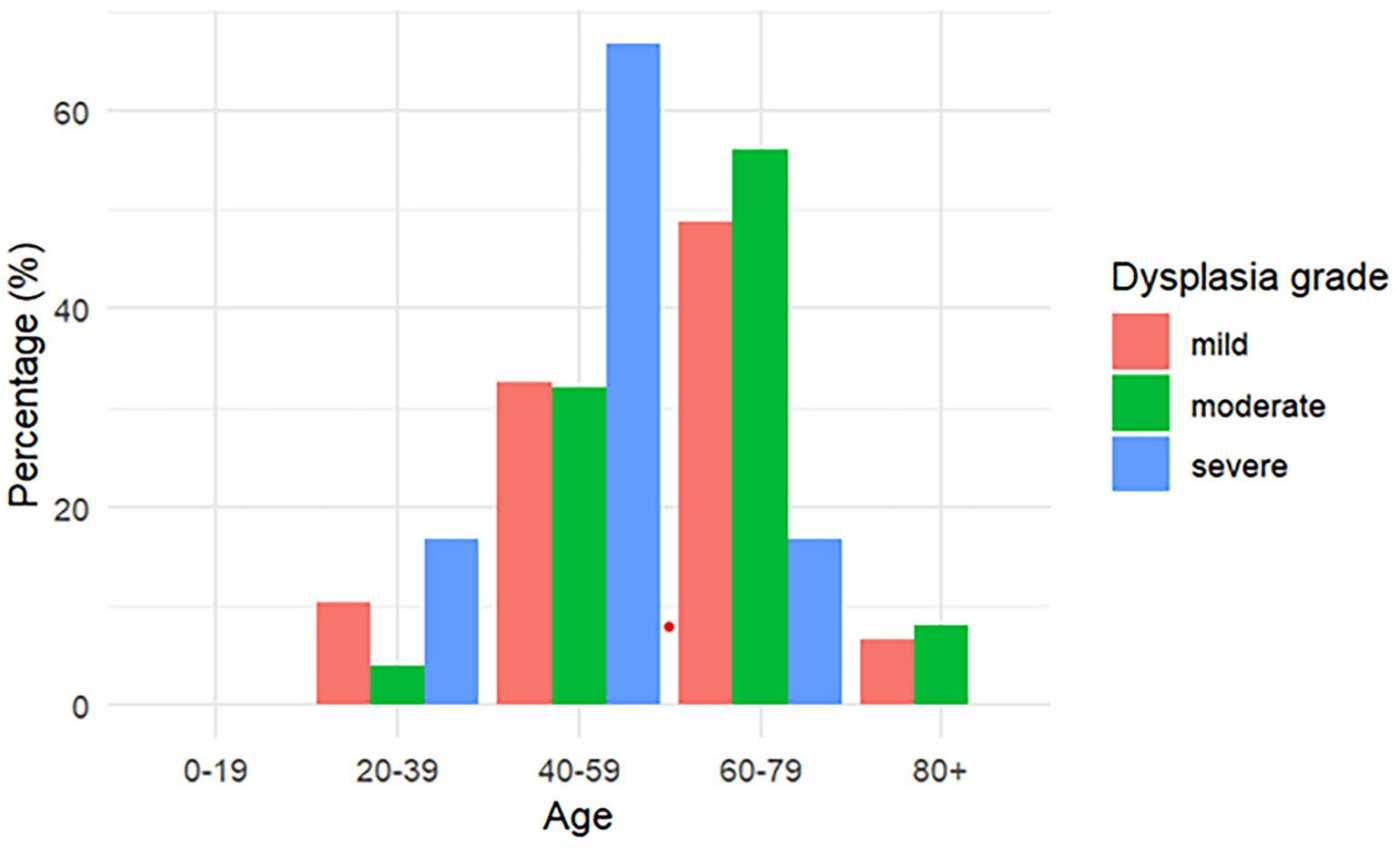
Age distribution of patients with oral epithelial dysplasia. [Color figure can be viewed at wileyonlinelibrary.com]

A total of 225 patients were diagnosed with OED during the study period (Table 1). Patient mean age was 60.0 years (range 22–97 years) (Figure 1) and 51.6% were females. Of the patients, 33.9% (*n* = 30) were healthy, meaning they had no comorbidities. Tobacco product use and alcohol consumption information were available for 34.7% (*n* = 78) and 10.2% (*n* = 23) of the patients, respectively. Of these patients, 26.7% were current or former smokers. Seven (3.1%) patients were snuff users. Altogether, 18% of the patients used alcohol.

**TABLE 1:** Characteristics of patients with oral epithelial dysplasia.

	*N*	%
Sex (*n* = 225)		
Males	109	48.4
Females	116	51.6
Age at the time of oral epithelial dysplasia diagnosis (*n* = 225)		
0–19 years	0	0
20–39 years	22	9.8
40–59 years	77	34.2
60–79 years	111	49.3
80– years	15	6.7
Smoking (*n* = 78)		
Yes	60	76.9
No	18	23.1
Alcohol consumption (*n* = 23)		
Yes	18	78.3
No	5	21.7
Biopsy location (*n* = 225)		
Mobile tongue	57	25.3
Gingiva	19	8.4
Hard palate	13	5.8
Buccal mucosa	43	19.1
Floor of the mouth	6	2.7
Lip mucosa	4	1.8
Oral mucosa, not defined	83	36.9
Grade of oral epithelial dysplasia (*n* = 225)		
Mild	193	85.8
Moderate	26	11.6
Severe	6	2.7
Size of oral epithelial dysplasia lesion (mm) (*n* = 62)		
0–4	8	13.1
5–9	16	29.5
10–14	17	27.9
15–19	7	11.5
20–24	9	14.8
25	5	8.2

### 3.2 Lesion Size and Histopathological Diagnosis

The size of the lesion in question was available in 62 cases. The median size was 11 mm (range 2–40 mm). The most common diagnosis was mild dysplasia, representing 85.8% (*n* = 193) of the cases (Table 1). Out of females, 83.6% had mild, 13.8% had moderate, and 2.6% had severe OED, while the corresponding levels for males were 88.1%, 9.2%, and 2.8%, respectively. Figure 1 presents the age distribution of the OED patients.

### 3.3 Malignant Transformation

**FIGURE 2:**
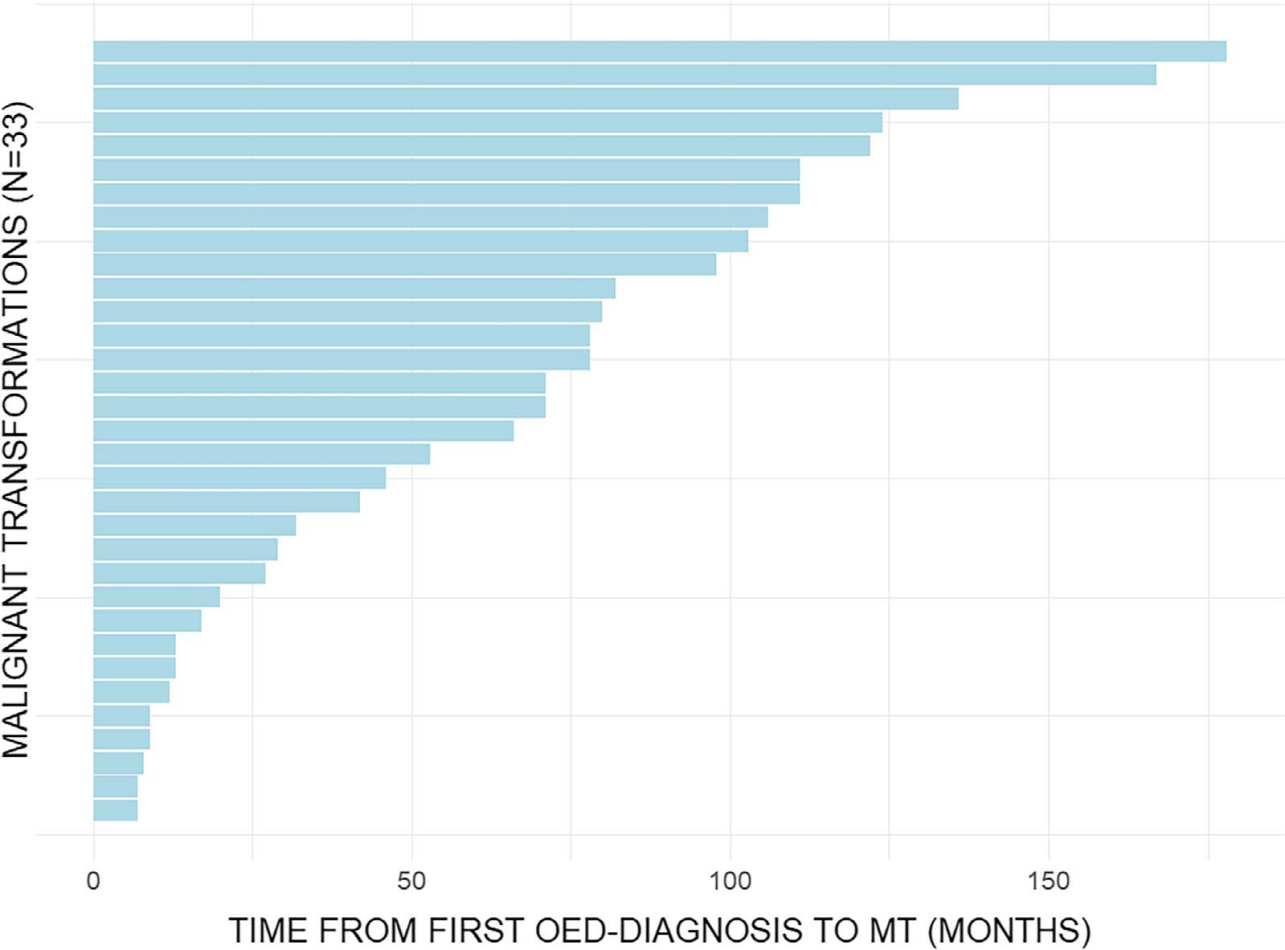
The time for malignant transformation (MT) to occur after the first oral epithelial dysplasia (OED) diagnosis (*N* = 33). [Color figure can be viewed at wileyonlinelibrary.com]

**FIGURE 3:**
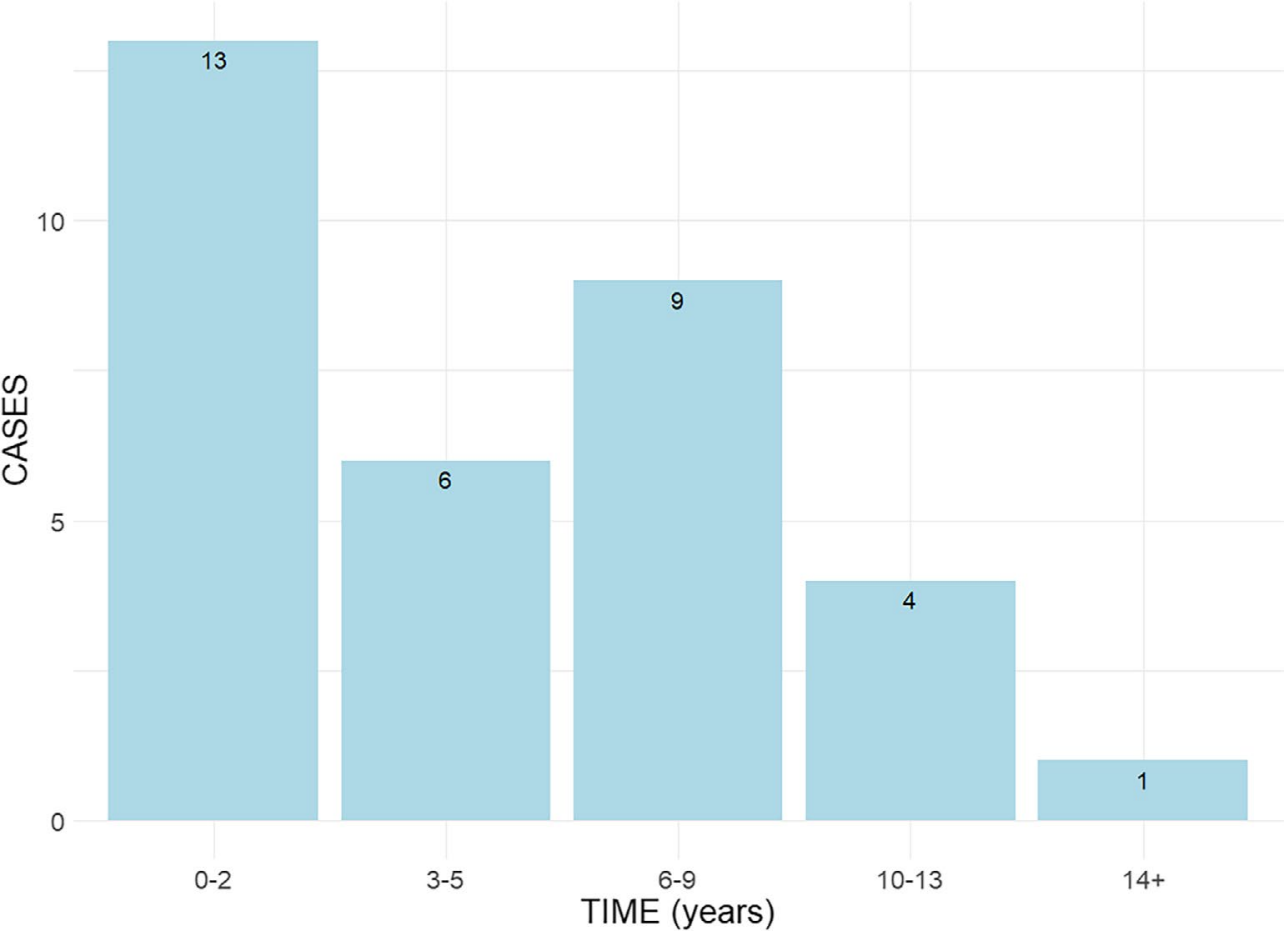
The time for MT to occur after the first OED diagnosis. [Color figure can be viewed at wileyonlinelibrary.com]

**FIGURE 4:**
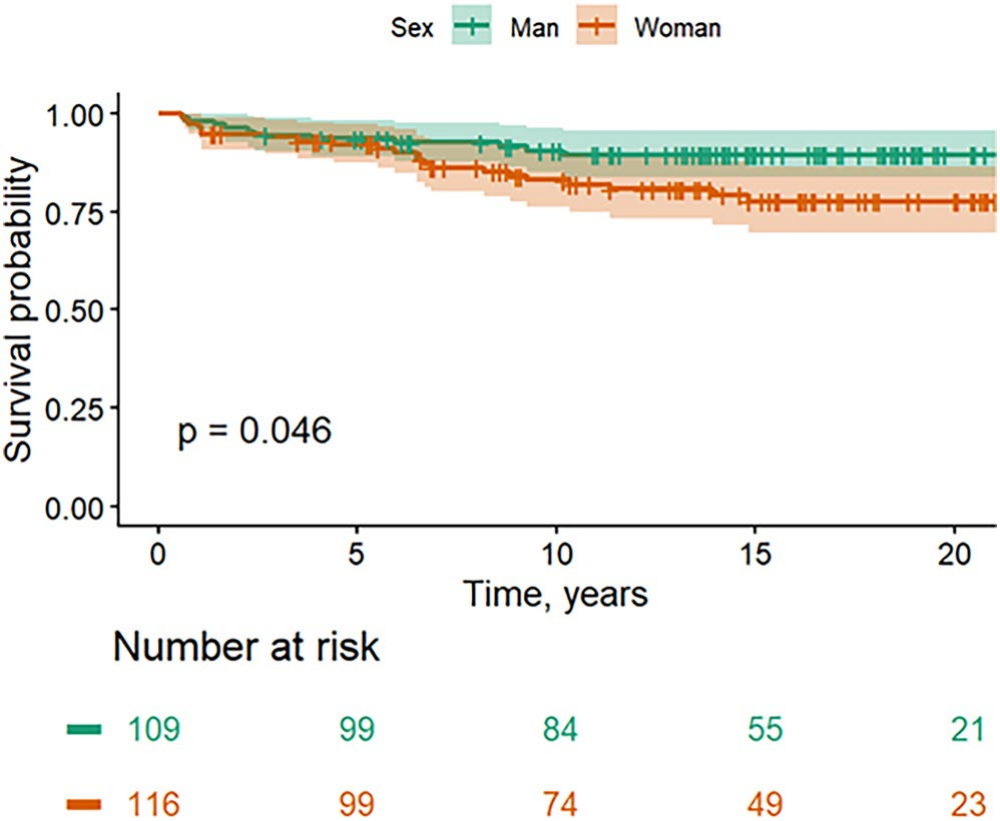
Kaplan–Meier curve showing slightly lower long‐term survival in females compared with males, with a statistically significant difference over the follow‐up time (log‐rank *p* = 0.046). [Color figure can be viewed at wileyonlinelibrary.com]

**FIGURE 5:**
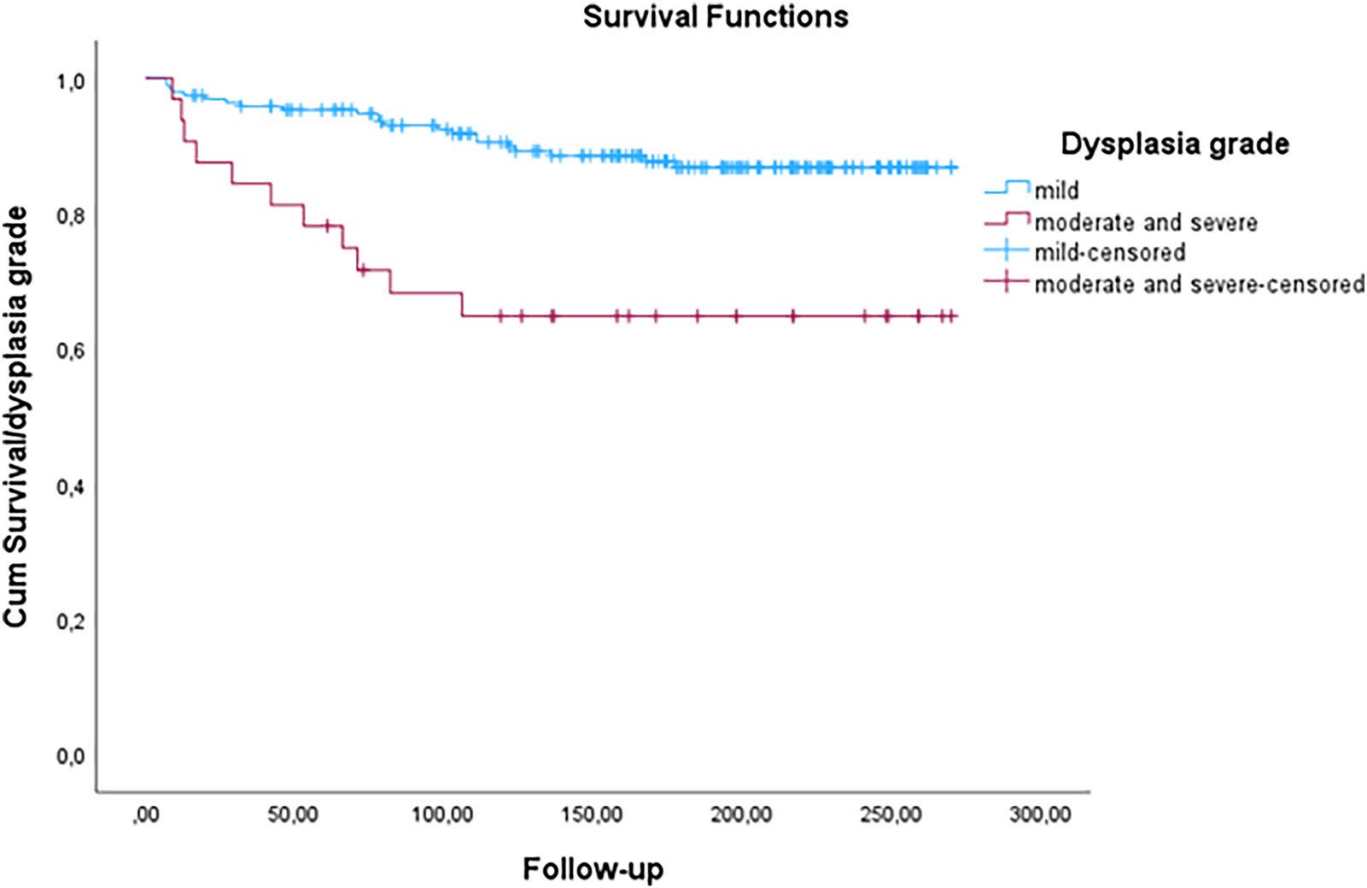
Kaplan–Meier survival curves of 225 patients with mild (upper, blue) or moderate and severe (lower, red) OED (*p* < 0.001, HR 3.5, 95% CI: 1.7–7.2). [Color figure can be viewed at wileyonlinelibrary.com]

**FIGURE 6:**
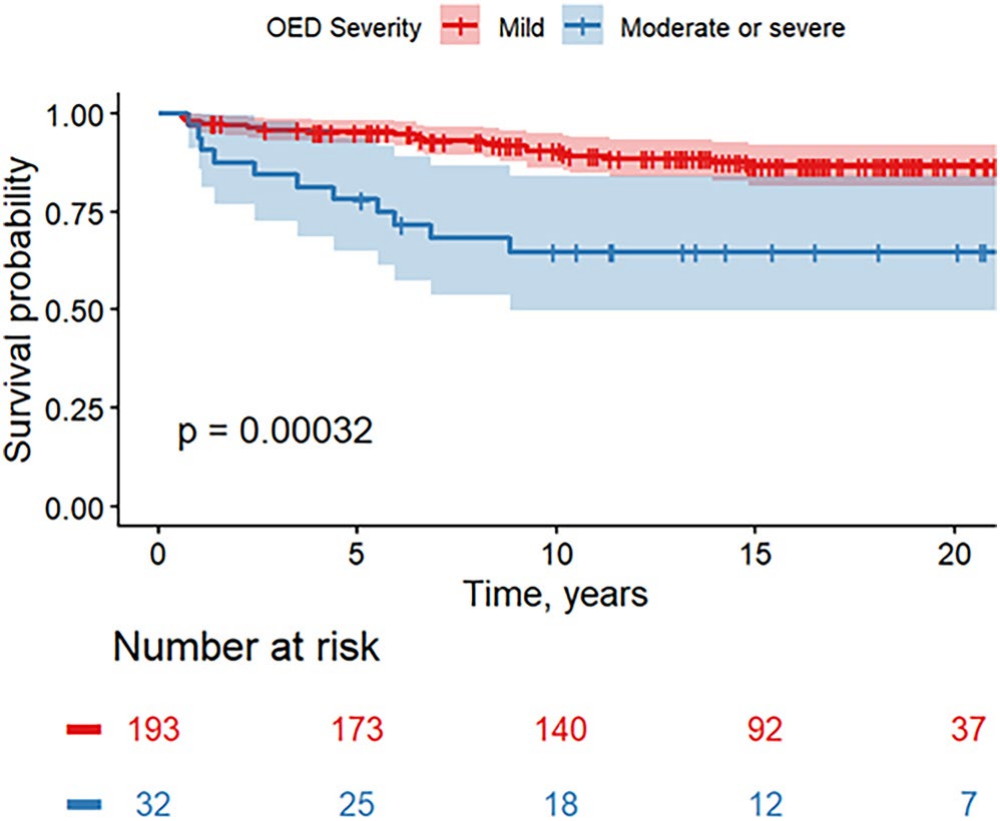
Kaplan–Meier survival probability in patients with moderate or severe dysplasia compared to mild dysplasia over the follow‐up time. [Color figure can be viewed at wileyonlinelibrary.com]

The mean follow‐up time was 13.5 years. MT occurred in 14.7% of the patients (*n* = 33), with a 5‐year cumulative incidence of 7.2% (95% CI: 4.5%–11.5%) (Figures 2 and 3). Of these patients, 66.7% were females and 33.3% were males. The 5‐year cumulative incidence of MT was 7.9% (95% CI: 4.2%–14.6%) in females and 6.5% (95% CI: 3.1%–13.1%) in males. In the log‐rank test for the difference in survival distributions, the difference between sexes was statistically significant, showing that females had lower survival probability than males did (*p* = 0.046, Figure 4). However, sex was not a significant predictor in a univariate Cox regression for MT‐free survival (*p* = 0.051, HR = 2.05, 95% CI: 0.996–4.24). However, at 10 years, the survival in females and males was 83.0% (95% CI: 74.3%–89.0%) and 90.5% (95% CI: 83.1%–94.8%), respectively, indicating a larger incidence of MT for females. Of the total 33 cases of MT, 17 occurred after the 5 years of follow‐up. The 5‐year cumulative incidence of MT for cases with mild OED was 4.8% (95% CI: 2.5%–8.8%) and 26.9% (95% CI: 11.1%–40.5%) for moderate OED. Of the six severe OED cases, MT occurred in two cases at 5.9 and 6.8 years, respectively. Due to the limited number of severe OED cases, there was insufficient statistical power to analyze the difference in OSCC development for moderate and severe OED independently. Therefore, moderate and severe OED samples were combined for the statistical analysis. The distribution of transformation‐free survival times differed statistically significantly between cases with mild OED and moderate and severe OEDs (log‐rank *p* < 0.001, Figures 5 and 6). Univariate Cox regression analysis showed an HR of 3.5 [95% Cl: 1.69–7.19] for cancer development when comparing mild OED to combined moderate and severe OED. Age and risk habits were not statistically significantly associated with MT time.

## 4 Discussion

In this retrospective study, we show the characteristics for MT of OED in a 10‐year period of 2001–2011 in southern Finland, in the hospital district surrounding the capital of Finland, Helsinki. This hospital district covers 1.78 million Finnish inhabitants, which is 31.6% of all Finnish inhabitants. MT occurred in 14.7% of the patients in a median time of 5.5 years (IQR 1.7–12.5 years). Dysplasia severity (*p* < 0.001) associated with an increased HR for cancer development. Age at the time of OED diagnosis or sex was not statistically significant factors for MT of OED.

Median age at the time of first OED (60 years) and female predominance in our data match the literature [11, 12, 13, 14]. We expected a higher prevalence regarding alcohol (18.0%) and tobacco consumption (26.7%) [6, 12]. However, when only considering the referral letters that contained information about smoking or alcohol use (*n* = 78), the prevalence of smoking increased, meeting our assumptions, while alcohol use still remained low (alcohol 17.3%, tobacco 76.9%). This may be because recording alcohol and tobacco habits has possibly not been considered essential anamnestic information at the beginning of the millennium. It would have been interesting to analyze the effect of smoking or alcohol consumption on the MTs, but this was not possible due to the limited data on those factors. The data regarding an association between Scandinavian snuff use and the risk of OSCC show an unclear association [3, 15]. However, snuff is known to cause oral mucosal lesions such as oral leukoplakia [16]. Snuff use data were only available for seven patients in our study, which may be related to our register study design with material from years 2001–2011. Then and continuing today, selling snuff is prohibited in Finland [3].

Globally, the MT rate (all dysplasia grades combined) varies between 8% and 18% [17, 18]. A pooled MT rate of OED has been reported as being slightly higher in Europe (12.6%) compared with North America (9.9%) and Asia (8.9%) [17]. The overall combined MT rate in the study was 10.5%. Different study designs and regional habits may explain the difference geographically. In this study from southern Finland, the MT rate was slightly higher than in southwestern Finland [19]. This could be due to differences in habits, socioeconomic factors, or differences in access to healthcare services or other demographic factors such as higher levels of pollutants.

Severity of OED has been established as a risk factor for MT in several studies [12, 19, 20, 21, 22]. Even mild dysplasias and lesions without dysplasia can carry a risk of MT [23]. In previous Finnish studies, the likelihood of MT was related to OED severity, and the risks for mild, moderate, and severe OED were 8.1%, 16.1%, and 38.5%, respectively [12, 19]. In general, studies have not shown sex to be associated with the risk of MT [14, 24]. By contrast, a few studies have shown a higher risk for MT in females compared with males [13, 25]. Age has not been regarded as an independent risk factor for MT [13]. However, molecular changes in OED accumulate during life. According to Iocca et al., with a study population of 15 000 OPMD patients, the mean age at the time of first OED diagnosis was 55 years [22]. In this study, the mean age was 60 years at the time of first OED diagnosis. This is in line with the accumulating molecular changes in OED. Females were more prone to MT than males were and had a slightly higher prevalence regarding OEDs.

The time for MT after the first OED diagnosis has been reported to vary between 2 and 5 years in most cases [18, 21, 22, 26, 27, 28, 29, 30, 31, 32, 33, 34]. However, malignancy may occur much later. In the current study, moderate OEDs were the fastest to transform into OSCC, in 2.5 years, while the average time was over 6 years for mild and severe OEDs. Our results are in line with the median transformation times of other studies from Finland (5.2–5.6 years) [12, 19]. Higher dysplasia grades have been associated with shorter transformation times [11, 27, 28, 35].

Interestingly, the overall MT time was slightly higher in the current study compared with previous studies globally [18]. Differences in time for MT in studies could be due to geographical factors, study data, and follow‐up time [33]. The time for MT was shorter for males than for females. In this study, sex was a borderline factor (log‐rank *p* = 0.046, Cox regression *p* = 0.051). There is no consistent evidence in other studies that sex correlates with transformation time [12, 19, 28, 29].

Similarly to other studies, we found the mobile tongue to be the most common site for OED [11, 12, 13, 19, 32]. OED on the lateral mobile tongue and floor of the mouth has been reported as high‐risk sites for MT in several studies globally [13, 31, 33, 35]. Ellonen et al. found a higher risk for shorter MT time in females in southwestern Finland, similarly to our results [19]. The median size of an OED lesion was 11 mm, which means that the lesions were discovered quite early.

The strength of this study is the large population‐based cohort from southern Finland. All cancer specimens in the hospital district were registered in the QPati patient database used in this tertiary care center in the Helsinki Metropolitan area, and therefore, this registry‐based study very efficiently covers the people diagnosed with OED in the region's population. The follow‐up time of the study was long (13.5 years, range 0.6–22.5 years). Adding clear exclusion criteria helped avoid concurrent malignancies. However, retrospective and registry‐based studies experience limitations with data collection. Therefore, some statistical analyses could not be performed since the information regarding, for example, tobacco and alcohol use was limited. Also, the original histopathological diagnoses were not re‐evaluated, causing possible variability in sample grading. On the other hand, this is the situation in real life. The last limitation of this retrospective study was the lack of treatment information concerning the dysplastic lesion. If available, we could have analyzed possible differences between the wait‐and‐see approach compared with active surgical treatment of MTs.

## 5 Conclusions

As a conclusion, 14.7% of OED cases transformed into OSCC, with a median time of 5.5 years. The severity of OED is a significant predictor of MT.

## Funding

The authors have nothing to report.

## Ethics Statement

The study protocol was approved by the Helsinki University Hospital area in southern Finland (HUS/124/2023) and HUS Regional Medical Research Ethics Committee (6349/2024). No patient consent was needed.

## Conflicts of Interest

The authors declare no conflicts of interest.

## Data Availability

The data that support the findings of this study are available on request from the corresponding author. The data are not publicly available due to privacy or ethical restrictions.
